# Informal rural healthcare providers in North and South India

**DOI:** 10.1093/heapol/czt050

**Published:** 2014-07-07

**Authors:** Meenakshi Gautham, K M Shyamprasad, Rajesh Singh, Anshi Zachariah, Rajkumari Singh, Gerald Bloom

**Affiliations:** ^1^London School of Hygiene and Tropical Medicine, London, UK, ^2^Centre for Research in New International Economic Order, Chennai, India, ^3^Garhwal Community Development and Welfare Society, Tehri Garhwal, Uttarakhand, India and ^4^Institute of Development Studies, Brighton, UK

**Keywords:** Informal providers, rural, India, healthcare, knowledge, Uttarakhand, Andhra Pradesh, health markets

## Abstract

Rural households in India rely extensively on informal biomedical providers, who lack valid medical qualifications. Their numbers far exceed those of formal providers. Our study reports on the education, knowledge, practices and relationships of informal providers (IPs) in two very different districts: Tehri Garhwal in Uttarakhand (north) and Guntur in Andhra Pradesh (south). We mapped and interviewed IPs in all nine blocks of Tehri and in nine out of 57 blocks in Guntur, and then interviewed a smaller sample in depth (90 IPs in Tehri, 100 in Guntur) about market practices, relationships with the formal sector, and their knowledge of protocol-based management of fever, diarrhoea and respiratory conditions. We evaluated IPs’ performance by observing their interactions with three patients per condition; nine patients per provider. IPs in the two districts had very different educational backgrounds—more years of schooling followed by various informal diplomas in Tehri and more apprenticeships in Guntur, yet their knowledge of management of the three conditions was similar and reasonably high (71% Tehri and 73% Guntur). IPs in Tehri were mostly clinic-based and dispensed a blend of allopathic and indigenous drugs. IPs in Guntur mostly provided door-to-door services and prescribed and dispensed mainly allopathic drugs. In Guntur, formal private doctors were important referral providers (with commissions) and source of new knowledge for IPs. At both sites, IPs prescribed inappropriate drugs, but the use of injections and antibiotics was higher in Guntur. Guntur IPs were well organized in state and block level associations that had successfully lobbied for a state government registration and training for themselves. We find that IPs are firmly established in rural India but their role has grown and evolved differently in different market settings. Interventions need to be tailored differently keeping in view these unique features.

**KEY MESSAGES**

- Informal providers (IPs) are an important source of primary health care for rural and poor households, but their roles have evolved differently in the two study sites.
- IPs in Tehri and Guntur differed with respect to years of education, modes of practice, relationships with the formal sector and levels of self organization.
- More than two-thirds of IPs at both sites knew how to manage common conditions, but they still prescribed/dispensed more drugs than necessary, especially in Guntur.
- Interventions with IPs need to take into account the specific aspects of the organization of health markets in different localities.

## Introduction

In India, as in many other low and middle-income countries, informal providers (IPs) deliver a substantial proportion of health care to rural, poor and underserved populations (Bloom *et al.* 2011; Sudhinaraset *et al.* 2013). This is largely a response to the relative unavailability of trained public and private sector health workers. The public sector provides health services to India’s rural population of over 800 million people (GOI 2011b), living in 640 867 villages through a limited network of 23 887 primary health centres (PHCs) and 4809 community health centres (CHCs) staffed by doctors, and 148 124 sub-centres staffed by auxiliary nurse midwives (ANMs) (GOI 2011a). The government defined the population and staffing norms for these facilities many years ago (Bhore 1946), but recent reports have concluded that they are inadequate to meet current needs (GOI 2005; HLEG 2011). Furthermore, the staffing of many facilities with trained health workers does not even meet these norms (Satpathy 2005). The ANMs based at the sub-centres are not sufficiently trained or empowered with drugs and commodities to provide a comprehensive package of care as the health department emphasizes mainly preventive maternal and child health functions for them (Mavalankar *et al.* 2010; George 2010). The same applies to the new cadre of accredited social health activists (ASHAs), who are trained under the National Rural Health Mission to link women and children with the health system for immunizations, institutional deliveries and antenatal and post-natal care, but not to deliver clinical care. 

India has a large private sector, with more healthcare providers than the public sector, but they mostly practice in urban, well-to-do areas (GOI 2005; De Costa and Diwan 2007). These factors have led rural and poor urban households to rely, to a large extent, on informal biomedical practitioners who provide modern medical care (Rohde and Vishwanathan 1995), and various types of traditional and folk healers (Sheehan 2009; Gautham *et al.* 2011; George and Iyer 2013). The former, also referred to as village doctors, village practitioners or Registered Medical Practitioners (RMPs), is the largest category of IPs (Deshpande *et al.* 2004; De Costa and Diwan 2007; Das *et al.* 2012). They are the most frequent first port of call for rural residents seeking health care (Gautham *et al.* 2011; George and Iyer 2013). A survey in Madhya Pradesh (De Costa and Diwan 2007) for example, enumerated 24 807 qualified doctors and 89 090 IPs. Seventy seven percent of the qualified doctors worked in urban areas and over 90% of IPs worked in rural areas. Another study of health seeking behaviour in the same state reported that 65% of practicing providers had no medical qualification and 70% of health seeking visits by rural households were to informal private providers (MAQARI 2011).

People consult IPs for a variety of common conditions, which include fevers, diarrhoea and respiratory problems (Rohde and Vishwanathan 1995; Kanjilal *et al.* 2007; Gautham *et al.* 2011; George and Iyer 2013) postpartum morbidity, anaemia and white discharge in women (Rao 2005; Tuddenham *et al.* 2010) and newborn illnesses (Kaushal *et al.* 2005). These providers tend to be viewed as a homogenous group, but studies in different settings suggest that they differ, in terms of education and training, the contents of their practice and their business model (Gautham *et al.* 2011; George and Iyer 2013). They work in complex health markets and are influenced by many factors, which include consumer expectations, relationships with other providers and formal and informal rules (George and Iyer 2013; Bloom *et al.* 2013). There is little systematic information on inter-regional differences in the markets for IPs in India. This paper reports the findings of an exploratory study aimed at bridging this gap. The study sought to map and characterize IPs in two different rural settings in the north and south of India, with respect to their education and training, practice characteristics and patient profile, knowledge and performance, treatment costs, and relationships with the formal sector. Private informal biomedical providers were the primary focus of this study. We refer to these as IPs in this paper.

## Methods

### The study areas

The study was located in Tehri Garhwal district in the north and Guntur district in the south. Tehri Garhwal is one of the 13 districts in Uttarakhand, a hilly state in the Central Inner Himalayan region. Guntur is one amongst 23 districts in the state of Andhra Pradesh (AP), located in the state’s coastal region. The districts were purposively selected to provide two very different contexts for this study. We had strong local contacts in both districts, which allowed us to optimize time and money resources.

Table 1 provides information on the two districts. Both are predominantly rural, but a larger proportion of the population is classified as rural in Tehri. Tehri also has a lower population density, smaller villages, and the density of rural roads is lower in Uttarakhand, Tehri’s parent state. Rural AP has a higher density of roads, but lower per capita monthly expenditure, and adult literacy is lower in Guntur than in Tehri. Tehri has a lower proportion of people in Scheduled Castes and Scheduled Tribe (SCs/ST) groups, who are amongst the most impoverished social groups in India. There are interesting differences in the districts’ health-related parameters. The average population per PHC and CHC is much larger in Guntur (42 530) than in Tehri (16 182). Guntur has three medical colleges within the district, while Tehri has none. Despite higher literacy rates and better coverage by government health facilities, the infant mortality rate at the time of this study was higher in Tehri.

**Table 1:** Guntur and Tehri: key social, demographic, economic, and health indicators

Indicators	Guntur	Tehri	All India
Total population (GOI 2011b)	4 889 230	616 409	1 210 193 422
Population density (population/surface area)	429/km^2^	151/km^2^	368/km^2^
% of population rural (GOI 2011b)	66.11	86.63	68.84
% of population Scheduled Castes (GOI 2001) (2001 census)	18.32	14.1	16.2 (2001 census)
% of population Scheduled Tribes (GOI 2001) (2001 census)	4.66	0.11	8.2
% of adults literate (GOI 2011b) (census 2011)	67.99	75.10	74.04
% of female adults literate (GOI 2011b) (census 2011)	60.64	61.77	65.46
No. inhabited villages (GOI 2001) (census 2001)	1047	1752	640 867
% of villages with population size ≤500 (GOI 2001)	1.45	82.4	39.75
State average monthly per capita expenditure (MPCE)—rural (NSSO 2010)	816 (for Andhra Pradesh)	901 (for Uttarakhand)	772
State rural road density (length) per 1000 km^2^ (GOI 2010a)	1225.36 (for Andhra Pradesh)	718.20 (for Uttarakhand)	920.49
Infant mortality rate (IMR)^a^	49	61	47
No. functioning PHCs (GOI 2011a)	64	28	23 887
No. functioning CHCs (GOI 2011a)	12	5	4809
Rural population per PHC/CHC	42 530	16 182	34 877
No. medical colleges (MCI 2013)	3	None	355

*Notes***:** ^a^IMR source for all India is the Sample Registration Survey, 2011 (GOI 2011c). This provides state and national level but not district level estimates. IMR source for Tehri is the Annual Health Survey, 2011 (GOI 2010–11). IMR source for Guntur is the estimate provided by the NFHS 2005 (IIPS 2008), and SRS 2010 (GOI 2010b) data for different regions of AP (both sources provided the same estimate).

### Sampling procedures

#### Blocks

A block is the smallest administrative unit in a district. We included all nine in Tehri and selected a sample of the 57 blocks in Guntur by stratifying them into three clusters by level of development (low, medium and high), and drawing proportional samples from each cluster (3 blocks from the low, 5 from the medium, and 1 from the high development cluster).

#### Providers and patients

We carried out field work during August–October 2011. We mapped all IPs in the two study sites with the help of a local providers’ association in Guntur and interviews with key community informants at both sites. In Tehri, we used information collected by a previous study in 2004 and supplemented this with fresh enquiries from key informants. We randomly sampled 100 IPs in Guntur and 90 in Tehri (with an additional 10 to allow for dropouts) for detailed enquiry into their knowledge and performance. These sample sizes were sufficient for an expected provider level outcome (such as quality of care) of 10% with a precision of 2% at the 95th level of confidence. The samples were random but drawn in proportion to the total numbers of IPs in each block cluster. 

Our study included observations of the interactions between providers and patients to evaluate provider performance. We recruited the first three consenting patients for the three most commonly presenting health problems at IP clinics—fever, diarrhoea and respiratory problems (total of nine patients per provider). Previous research using observations (Chakraborty and Frick 2002) suggests an optimum number of four observations per provider to allow for within-provider variations. However, due to time and financial constraints we had to limit the observations to three per condition, thereby yielding nine observations per provider.

### Study measures and data collection processes

We used quantitative and qualitative methods to obtain data on a wide range of study variables:

#### Provider education, training and practice characteristics

We used a structured questionnaire to interview all the IPs we could identify and map. Providers were asked about their years of schooling, training and apprenticeships, details of practice, most common conditions seen, system of medicine practiced, source of new knowledge, referral relationships and membership of any association. We also enumerated all formally trained and registered doctors in public and private health facilities in the study areas after confirming their availability from local residents.

#### Provider knowledge

We developed a knowledge assessment questionnaire to assess the knowledge of IPs on management of fevers, diarrhoea and respiratory problems. These three conditions were selected because they commonly present at the primary level and are amongst the most common ones that IPs treat (Gautham *et al.* 2011). Our team of three physicians (a paediatrican, a general physician and a cardiologist) reviewed the World Health Organization’s protocols for management of these conditions (GOI 2009; WHO 2009). They drew on these protocols to develop questions about the knowledge of the providers on history taking, physical examination and treatment of patients presenting with these conditions. Each question was scored on the basis of the number of correct responses. We interviewed the subset of 100 IPs in Guntur and 90 in Tehri.

#### Provider performance

We developed a patient–provider observation tool to document providers’ technical quality of care for each of the three conditions. The tool included history taking, physical examination, providers' diagnosis and treatment provided. These corresponded with most sub-items in the knowledge questionnaire. For example, in the knowledge questionnaire providers were asked about the physical examination they would perform on a diarrhoea patient and given a score of 1 if they named an appropriate check for dehydration in an adult or child. During the observation, they received a score of 1 if they correctly examined a diarrhoea patient for dehydration. All dispensed and prescribed medicines were noted through the tool. This tool was used to observe the subset of 100 sampled IPs in Guntur and 90 in Tehri (the process is described in the last paragraph in this subsection).

#### Patient profile

We used a patient exit interview to determine patients’ income levels, sequence of care seeking for their present complaints, their satisfaction with the current provider and his services, accessibility of the current clinic, and total costs incurred. This tool was used only with the patients that were observed in interactions with the providers. We used these data to provide a summary profile of the patients. We also used them to report on the costs of treatment.

#### Relationships with the formal sector

We used an in-depth interview questionnaire for providers to explore market related factors associated with IPs’ practices, especially their relationships with formal sector doctors, public and private. We also drew upon some quantitative data from the mapping survey on sources of IP knowledge and referrals in our final reporting of these relationships. 

Each tool was translated into Hindi and Telugu and piloted with five (non-sample) IPs before being used.

We first mapped and interviewed all the IPs in the study areas and then drew our provider samples from the mapped data. One field investigator was stationed at each sampled (and consenting) provider clinic for up to 3 days. He/she was required to build rapport with the IP, repeat the research objectives and processes for the provider’s full and complete understanding, and administer the in-depth interview guide and the knowledge assessment, to begin with. While at the clinic, the investigator waited for spontaneously presenting patients at the clinic: three each for fever, diarrhoea and respiratory problems. With the IPs’ help in gaining initial consent, the investigator sought the patient’s full consent for observing the interaction, unobtrusively. After each observation, the investigator interviewed the patient outside the clinic.

### Ethical clearance

The implementing organization, Crenieo, has an Ethical Review Committee of senior academics from Madras University. They reviewed the study and provided ethical approval.

### Analysis

We used stata (versions 8 and 9) for the quantitative analysis and manual qualitative techniques for the in-depth interviews and all open ended data. Quantitative analysis of the structured interview data included means and frequency distributions. We analysed the patient observations dataset by calculating the means per condition (average across all three patients of that condition), per provider, and then the means for each site.

## RESULTS

### Distribution of IPs and formally trained doctors

**Figure 1:**
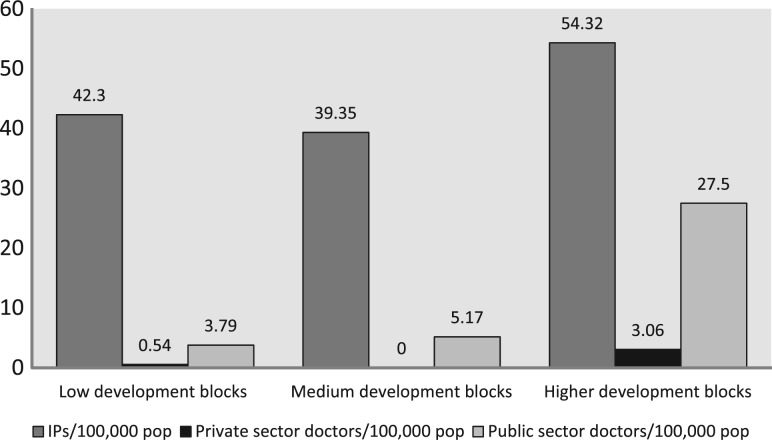
Tehri: available informal providers, private sector doctors and public sector doctors per 100 000 population across the low, medium and high development block clusters

**Figure 2:**
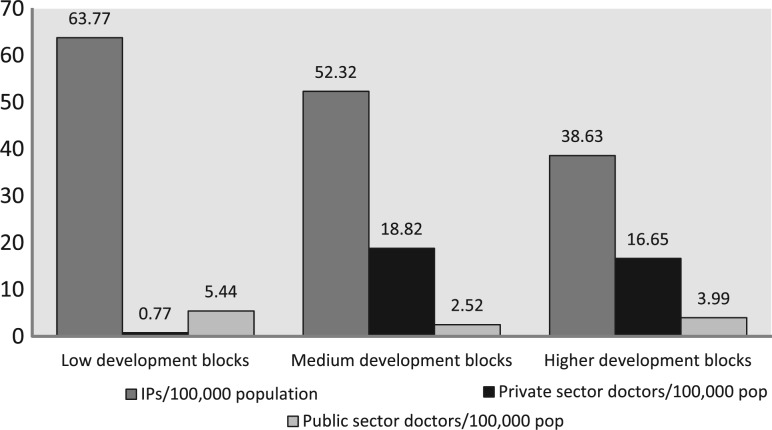
Guntur: available informal providers, private sector doctors and public sector doctors per 100 000 population across the low, medium and high development block clusters

We mapped 368 IPs in Guntur and 263 in Tehri (Table 2). We also mapped 63 formally trained and certified biomedical doctors in Tehri (58 public; 5 private) and 132 in Guntur (24 public; 108 private). The ratios of IPs and professional doctors to the general population were 1:2299 and 1:9599, respectively, in Tehri, and 1:1941 and 1:5412 in Guntur. Figures 1 and 2 show the availability of IPs and doctors per 100 000 population in low, medium and high development blocks. In Tehri (Figure 1), the majority of public and almost all the private sector doctors were concentrated in the high development blocks, while the low and medium blocks were served mainly by IPs. However, the largest number of IPs per 100 000 population was also present in the high development blocks. In Guntur (Figure 2), the largest number of IPs per 100 000 population was available in the low development blocks. Also, in Guntur, private doctors were present in large numbers in both high and medium development blocks, and exceeded the density of public sector doctors. In contrast, private doctors were rare in Tehri. We found 5 private doctors and 58 public sector doctors in Tehri, but the majority of public doctors were in the high development blocks.

**Table 2:** Distribution of informal providers and formal providers (qualified doctors) in different block clusters in the study districts

District	Block clusters ranked by selected development indicators	Population (2001 census)	No. informal providers	No. private doctors	No. public sector doctors	Ratio of informal providers and population	Ratio of private doctors and population	Ratio of public doctors and population	Total no. qualified doctors	Ratio of all doctors and population
Tehri	Low (two blocks)	184 320	78	1	7	**1:2363**	**1:184 320**	**1:26 331**	8	**1:23 040**
	Medium (five blocks)	289 730	114	0	15	**1:2541**	**na**	**1:19 315**	15	**1:19 315**
	High (two blocks)	130 697	71	4	36	**1:1841**	**1:32 674**	**1:3631**	40	**1:3267**
Total	Nine blocks	604 747	263	5	58	1:2299	1:120 949	1:10 426	63	1:9599
										
Guntur	Low (three blocks)	128 572	82	1	7	**1:1568**	**1:128 572**	**1:18 367**	8	**16 072**
	Medium (five blocks)	435 630	228	82	11	**1:1911**	**1:5313**	**1:39 603**	93	**1:4684**
	High (one block)	150 125	58	25	6	**1:2588**	**1:6005**	**1:25 021**	31	**1:4843**
Total	Nine blocks	714 327	368	108	24	1:1941	1:6614	1:29 764	132	1:5412

### IP’s background: education and training

Almost all the IPs were male (97% in Tehri and 98% in Guntur) with a mean age of 39 years in Tehri and 42 in Guntur. The majority were in their 30s and 40s, but around a quarter were younger than 30 (25% in Tehri and 19% in Guntur) and had been in practice for 5 years or less.

We found marked differences in the education and training of IPs between the two districts (Table 3). In Tehri 94% had completed 11 or more years of schooling and 43% had graduated from college, compared with 41% and 10%, respectively, in Guntur. Ninety-three percent of Tehri IPs possessed a diploma or a certificate related to a health science, such as pharmacy, ayurvedic medicine and electrohomeopathy. The latter two were not obtained from a formal, certified institution. Only 35.6% of Guntur’s IPs held a diploma/certificate, and most of those had recently attended courses organized by the AP government’s ‘Community Paramedic’ training programme initiated in 2008. The majority of Guntur IPs had learned their trade through apprenticeships with qualified doctors. All the Guntur IPs had worked as a doctor’s compounder or assistant for a mean period of 7 years before setting up an independent practice. In Tehri, only 55% had served an apprenticeship with another provider.

**Table 3:** Education, training, knowledge and performance of informal providers in Tehri and Guntur

IPs’ educational and training background	Tehri	Guntur
**Education (Tehri *n* = 263; Guntur *n* = 368)**		
Completed 11 or more classes in school	246 (94%)	152 (41%)
Graduates	112 (43%)	35 (10%)
Held a health related diploma or certificate	243 (93%)	131 (35.6%)
**Apprenticeships (Tehri *n* = 263; Guntur *n* = 368)**		
Worked as compounder/assistant before starting independent practice	144 (55%)	368 (100%)
Worked under a qualified doctor (with MBBS or MD degrees)	106 (40%)	336 (91%)
Average number of years of apprenticeship	4 years	7 years
**Independent practice (Tehri *n* = 263; Guntur *n* = 368)**		
Mean years of independent practice in the present location	10.5 years (range 1 month–47 years)	13 years (range 1 month–50 years)
**IPs’ knowledge and management^a^ of diarrhoea, fever and respiratory conditions (Tehri *n* = 90; Guntur *n* = 100)**		
Diarrhoea: mean scores and percentages		
Knowledge (maximum score = 15)	11.46 (76.43%)	11.94 (79.60%)
Performance (maximum score = 14)	9.21 (65.79%)	10.19 (72.82%)**
Fever: mean scores and percentages		
Knowledge (maximum score = 13)	9.08 (69.88%)	8.91 (68.50%)
Performance (maximum score = 14)	6.07 (43.41%)	5.66 (40.47%)
Respiratory conditions: mean scores and percentages		
Knowledge (maximum score = 15)	10.15 (67.70%)	10.45 (69.67%)
Performance (maximum score = 15)	7.60 (50.66%)	8.33 (55.57%)*
All three conditions combined: mean scores and percentages		
Knowledge (maximum score = 43)	30.71 (71.42%)	31.3 (72.79%)
Performance (maximum score = 43)	22.88 (53.22%)	24.19 (56.27%)

*Notes*: ^a^IPs’ performance on illness management as evaluated using patient–provider observations. MBBS = Bachelor of Medicine, Bachelor of Surgery; MD = Doctor of Medicine.

**P < 0.005 (*T*-test *P* value for difference in performance between Tehri and Guntur), *P < 0.05.

### Characteristics of IP practices

A majority of IPs had strong local roots and long-established practices. More than half the IPs were born in the block where they practiced, or in the same district (Table 4). There were differences between the IP practices in the two districts. Tehri IPs were almost all clinic-based, whereas around 40% of IPs in Guntur provided doorstep services to their clients, routinely visiting them at their homes, and another 35% combined doorstep with clinic-based services. More than 90% of clinic-based IPs in both districts were available 7 days a week for an average of 9–11 h. In Guntur, mobile providers went on their rounds for an average of 5.31 h a day, covering 5–6 villages within a mean distance of 2.33 km. Most used bicycles (48%) or motorcycles (40%) to move around, and 12% went on foot. Guntur IPs reported larger catchment populations of 604 households on average, double the number reported in Tehri. Guntur IPs also reported higher daily patient loads of 17 patients on average, compared with 14 in Tehri.

**Table 4:** Details of informal providers’ practice characteristics in Tehri and Guntur

IP practice characteristics	Tehri	Guntur		
**Mode of practice**	***n* = 263**	***n* = 368**		
Mainly clinic-based	99.00%	25.54%		
Mainly mobile (routinely go on rounds)	0.50%	39.67%		
Clinic and mobile	0.50%	34.79%		
**Location of clinic (for those with clinics)**	***n* = 263**	***n* = 222**		
Clinic at IP's residence	29.28%	39.64%		
Mean distance of clinic from IP's residence (for clinics that are not in the residence)	4.31 km	2.10 km		
**Nativity and origin of the IP**	***n* = 263**	***n* = 368**		
Born in the same block	49.81%	52.99%		
Born in the same district	19.77%	41.03%		
Born in the same state	11.79%	5.71%		
Born in another state	18.63%	0.27%		
**Clinic operating hours (for those with clinics)**	***n* = 263**	***n* = 222**		
Open every day of week	90.00%	95.96%		
Min–max hours open/day	2–13 h	2–24 h		
Mean hours open/day	9.06 h	11.21 h		
**Mobile provider characteristics**	**NA**	***n* = 274**		
Min–max hours of travel/day	NA	1–13 h		
Mean hours of travel/day	NA	5.31 h		
Min–max distance covered/day	NA	1–15 km		
Mean distance covered/day	NA	2.33 km		
Mobile provider's transport	NA	Cycle: 48.18%	Motorcycle: 40.15%	Walk: 11.68%
**Clientele of informal providers**	***n* = 263**	***n* = 368**		
Average no. patients/day in the low illness season	10.62	11.43		
Average no. patients/day in the high illness season	16.52	22.59		
Average no. patients in the low and high illness seasons combined	13.57	17.01		
Min–max no. client households	15–2500	100–900		
Mean no. client households	365.83	603.45		
**Medical system adopted by the IP**	***n* = 263**	***n* = 368**		
Prescribes/dispenses only allopathic medicines	29.27%	94.29%		
Prescribes/dispenses only ayurvedic/homeopathic/unani medicines	1.52%	0.54%		
Prescribes/dispenses mixed medicines	69.21%	5.16%		
**Dispensing of medicines**	***n* = 263**	***n* = 368**		
Only dispensing	41.44%	17.39%		
Only prescribing	6.84%	45.38%		
Dispensing mostly but also prescribing	49.43%	26.36%		
Prescribing more but also dispensing	2.28%	10.87%		

The type of services provided differed between the two districts. IPs in Guntur used only allopathic (western biomedical) drugs, but many in Tehri (around 70%) supplied a blend of biomedical and non-biomedical drugs. Tehri IPs usually dispensed medicines at their clinics, whereas nearly half of Guntur IPs said that they only prescribed and another 11% said that they prescribed most drugs and dispensed a few of them.

IPs in Guntur were organized and united with 76% stating that they were members of their local ‘Mandal [block] RMP Association’. In Tehri only 18% were members of an association, usually linked to a local professional group such as pharmacists or electrohomeopaths. There was no local informal provider association in Tehri.

### IPs’ knowledge and performance

We assessed the knowledge and performance of the IPs in management of fevers, diarrhoea and respiratory conditions. They had similar levels of knowledge in both districts (Table 3), obtaining mean percentages in the knowledge assessment questionnaire of 71% in Tehri and 73% in Guntur. We observed 810 contacts between patients and IPs in Tehri and 900 in Guntur. We found that IPs in Guntur performed slightly better on management of diarrhoea and respiratory conditions. 

The major difference in illness management between the two sets of IPs was in the use of injections and antibiotics. In Guntur, 71% of patients received an injection, whereas in Tehri only 13% of patients received one. Less than 1% of patients were referred at either site. Guntur patients received a mean of 1.19 antibiotics and 30% patients received two or more antibiotics. Tehri patients received a mean of 0.94 antibiotics and 19% patients received two or more antibiotics.

### Profile of patients

Patient exit interviews revealed that this was the first provider visit for the present illness episode for 91% of Tehri patients and 82% of Guntur patients. The mean age of patients was similar at each site: 35 years in Tehri and 32 years in Guntur. A majority were adults at both sites (86% in Tehri and 78% in Guntur). More than 60% of the adult patients and 55% of the child patients were male. Patients differed in their affiliation to social groups. Seventy-five percent of patients in Tehri belonged to an upper caste. Around 17% belonged to SCs/STs and 9% were from other backward castes (OBCs). In Guntur, a larger proportion of patients were from OBC groups (43.56%) and from SCs/STs (31.78%). Less than 25% were from general/forward caste groups. Mean monthly household incomes (as reported by patients) were not too different across the two sites: INR 3995 in Tehri and 4271 in Guntur.

### Cost of treatment

Patients paid slightly more for their treatment in Tehri than in Guntur (INR 76 compared with 68). When we added the estimated prices of prescribed medicines that Guntur patients would need to purchase from a pharmacy, the total cost of treatment in Guntur turned out to be higher (INR 132) than in Tehri (INR 78).

Patients travelling to clinics (810 in Tehri and 553 in Guntur) had reached there in about half an hour on average in Tehri and 12 min in Guntur. A majority, 63% patients in Tehri and 58% in Guntur, had walked to the clinic; 0.12% in Tehri and 16.7% in Guntur had travelled on a cycle and 36% in Tehri and 25% in Guntur had travelled by motorized transport, such as a tractor, bus or jeep. In Guntur, 39% patients did not travel to any clinic as the provider visited them at home; 65% patients in Tehri and 82% in Guntur paid nothing for travel and those that did had low travel costs with a mean of INR 16.53 in Tehri and INR 13.12 in Guntur.

### IPs’ relationships with the formal sector

Interviews with IPs revealed that they had good interpersonal relations and interactions with private doctors in Guntur but not in Tehri. During the mapping survey, 40.5% of Guntur IPs said that they received commissions from private doctors for referrals, and 7% received gifts like small medical equipment and sample medicines. Doctors were also an important source of knowledge for Guntur IPs; 54% of them said that doctors were their only source of new knowledge and 8% included doctors as one of their sources (including representatives of drug companies and drug literature). IPs said that they received medical advice and learned about new treatment techniques and modern equipment from formally trained doctors. 

Monetary incentives were not the only reason that Guntur IPs referred to private doctors. They also had high levels of confidence and faith in these doctors and saw other mutual benefits.

We have good relation with qualified doctors. They advise us not to take risks and keep the patients with us. They allow us inside the operation theatre. They don’t let us treat, they only treat. When their staff are not available they allow us in. They give 10% per case.

I know 20 qualified doctors. They will give us suggestions regarding treatment sometimes. When I refer poor patients, they will give concession during diagnosis and treatment.

    —informal providers in Guntur

IPs in Guntur said that the government system did not provide good medical care and they referred only the poorest patients to government facilities. The same government doctors were their trainers through the state government sponsored training programme, and we found no overt hostility between the IPs and the public health system in the district. However, there was a latent perception of formal sector doctors as competitors. Guntur IPs said that as roads improved and people acquired more wealth, increasing numbers of patients consulted doctors in nearby towns.

We found a different situation in Tehri, where 96% of IPs said that they had negligible or no interactions with formal sector doctors. Since there were only five private doctors within the district, IP referrals were directed equally towards public facilities and private facilities, including private facilities in nearby towns outside the district. Referrals to government facilities were not just to the government hospital but also to primary level health centres where doctors were available. We also heard accounts of bitter experiences with state government officials in the health department, who demanded that IPs show their certificates and diplomas and sometimes demanded bribes.

## Discussion

Our study found several differences between the two districts in the education, training, and practices of IPs and their relationships with the formal, organized health sector. These may be related to the differences in the terrain and demography of the two districts and the availability of licensed public and private health service providers. Guntur is more densely populated, with more roads and many more private doctors. The majority of its IPs began their working life as employees of these doctors and maintained relatively close links with them. They mainly prescribed allopathic drugs and much of their practice was carried out through door-to-door visits. Tehri is mountainous, with fewer roads and a more widely dispersed population. It had very few private doctors and more public sector doctors, although the majority was in the better developed areas. Most of its IPs had some form of post-secondary school training. They all had clinic-based practices and dispensed both Western and traditional medicines.

There were marked differences between the two localities in the degree to which the IPs were organized and in their relationship with the state government. In Guntur, IPs have had a long history of membership in associations. These associations have grown in strength and in 2008 they reached agreement with the state government about the provision of training to their members, with the aim of certifying them through a state paramedical council. The government subsequently organized a training scheme for many IPs. In Tehri, the IPs were not united or organized and their relationships with doctors were neither strong nor mutually supportive. Moreover, the government of Tehri’s state was hostile towards IPs and state health authorities frequently harassed them as quacks. The explanation for these differences may be related to the different forms the health markets have taken in the two localities. In Guntur, the better road network and denser population may have enabled IPs to network more easily and set up practices in poorer areas, where they faced less competition from formal doctors. It may also have made it easier for private doctors to maintain links with IPs in the more remote villages as a source of referrals. There were many more private doctors in Guntur. This may reflect the presence of three medical colleges, two of which were private. There are no medical colleges in Tehri, and only a handful of private doctors. The mountainous terrain and poor roads may have hindered effective networking between IPs.

Our study confirmed the findings of several other studies (Rohde and Vishwanathan 1995; Gautham *et al.* 2011; George and Iyer 2013; Das *et al.* 2012) that IPs are a significant source of basic health care for rural residents. A study of IPs in two other Andhra districts, Warangal and Karimnagar, located in the state’s Telengana region, also reported ‘doorstep’ services (Gautham *et al.* 2011) by mobile IPs who travelled from house to house and village to village on their daily service delivery rounds. As in Guntur, private doctors were present in large numbers at the block level in these two Telengana districts (Warangal and Karimnagar), and here too there were ‘complementarities’ rather than ‘turf wars’ between formal providers and IPs (Gautham *et al.* 2011).

Although the public health community has gradually recognized the importance of IPs (Yadav *et al.* 2009), their future role is a matter of heated debate. Much of the Indian medical establishment views them as dangerous quacks and the courts in some states have ordered the government to shut their clinics (Express 2012). On the other hand, national policies framed by India’s Planning Commission in the 11th and 12th Five-Year Plans have called for the integration of IPs into the health system (GOI 2008, 2012).

According to India’s Constitution, the states are responsible for health services. This makes the recent initiative by the Government of AP to provide training to IPs and register them with the state’s paramedical board particularly important. At the time of our study, only around 20% of IPs in the study sites had been trained so we are unable to draw any conclusions about the training outcomes. The study has shed light on features of IP practice that need to be addressed in order to improve the quality of their services. They have a reasonable amount of knowledge on protocol-based management of fevers, diarrhoea and respiratory conditions, but they do not always apply this knowledge and they over-prescribe drugs, particularly in Guntur.

International evidence suggests that training, on its own, has only a modest impact on performance (Shah *et al.* 2011). Complementary supply-side interventions may include regular supportive supervision and changes in financial and non-financial incentives to encourage good practice. Demand-side measures may include public education to reduce information asymmetries between providers and patients and increase awareness of rational drug use. Also, the services provided by IPs could be included in some kind of financing scheme to reduce the cost of primary health care for the poor, through community-based insurance schemes or vouchers for example. However, this would raise many issues concerning the control of costs and the quality of care.

There are a few study limitations. As this was a scoping study, we did not attempt to analyse contextual predictors of differences in the IP markets at the two sites. Our objective was to build profiles of IPs across two very different sites and these now suggest that local contexts may strongly influence the way that IP markets evolve. This limits the generalizability of the findings to other contexts. In future, we would like to examine these factors systematically. Second, our assessment of IPs’ knowledge and performance was limited by the boundaries of the tools we employed. Our knowledge assessment tool was based on World Health Organization (WHO) protocols for health workers’ management of the most commonly presenting conditions at the primary level—fevers, diarrhoea, respiratory conditions—and so our findings reflect IPs’ knowledge about basic first level management of these conditions.

## Conclusions

Our study has shown that although IPs are on the margins of formalized medicine, over the years they have established important niches, particularly in rural areas. They work within well-developed institutional arrangements, which have evolved in different directions in different contexts. This finding dispels the myth that IPs are solo ‘quacks’ with only limited links to their community and to local institutions. It also underlines the likelihood that IPs will continue to play a role for quite a long time irrespective of increasing incomes and infrastructural development. Strategies for substantially increasing access by India’s rural residents to safe, effective and affordable health care will need to involve IPs. The case studies show that interventions aimed at integrating IPs into the health system and improving their performance need to take the specific characteristics of the local health market system into account.

## Authorship

M.G. led the research study and drafted the paper. K.M.S., R.S., A.Z. and R.S. contributed to the conception, design and implementation of the study, analysis and interpretation of the data, and provided critical intellectual inputs to the paper. G.P. contributed to structuring of the paper and provided critical intellectual inputs towards substantial revisions.

## Supplementary Data

Supplementary data are available at *Health Policy and Planning* Online.

## Funding

This study was funded by the Results for Development Institute, as a part of a Center for Health Market Innovations (CHMI) project to better understand informal providers in Asia and Africa. CHMI is funded by the Bill and Melinda Gates Foundation and the Rockefeller Foundation.

## Conflict of interest

None declared.
